# Characterization of Alpelisib in Rat Plasma by a Newly Developed UPLC-MS/MS Method: Application to a Drug-Drug Interaction Study

**DOI:** 10.3389/fphar.2021.743411

**Published:** 2021-11-25

**Authors:** Qiong Wang, Xia Lan, Zhuofei Zhao, Xiaohang Su, Yuji Zhang, Xiao-Yang Zhou, Ren-Ai Xu

**Affiliations:** ^1^ The Third Affiliated Hospital of Shanghai University, Wenzhou People’s Hospital, Wenzhou, China; ^2^ Chongqing University Cancer Hospital, Chongqing, China; ^3^ School of Basic Medical Sciences, Henan University of Science and Technology, Luoyang, China; ^4^ The Key Laboratory of Geriatrics, Beijing Institute of Geriatrics, Institute of Geriatric Medicine, Chinese Academy of Medical Sciences, Beijing Hospital/National Center of Gerontology of National Health Commission, Beijing, China; ^5^ The First Affiliated Hospital of Wenzhou Medical University, Wenzhou, China

**Keywords:** drug interaction, pharmacokinetics, CYP3A4 inhibitors, ultra-performance liquid chromatography–tandem mass spectrometry, alpelisib

## Abstract

Alpelisib, an oral selective and small-molecule phosphoinositide 3-kinase inhibitor, was lately approved in the United States to treat breast cancer. A sensitive method to quantify alpelisib levels in rat plasma on the basis of ultra-performance liquid chromatography–tandem mass spectrometry technique was established and validated, which was successfully employed to explore the effects of CYP3A4 inhibitors on alpelisib pharmacokinetics in rats. A C18 column named Acquity UPLC BEH C18 was applied to achieve the separation of alpelisib and internal standard duvelisib after protein precipitation with acetonitrile. The mobile phase in this study had two components, namely, acetonitrile and water having 0.1% formic acid, and a program with gradient elution method was used at a flow rate of 0.40 ml/min. Mass spectrometry in a positive multiple reaction monitoring mode was operated. In the scope of 1–5,000 ng/ml, this assay had excellent linearity. Our newly developed assay was verified in all aspects of bioanalytical method validation, involving lower limit of quantification, selectivity, accuracy and precision, calibration curve, extraction recovery, matrix effect, and stability. Then, this assay was used to detect the plasma levels of alpelisib from a drug-drug interaction investigation, where ketoconazole remarkably increased the plasma concentration of alpelisib and changed alpelisib pharmacokinetics more than itraconazole. This study will help better understand the pharmacokinetic properties of alpelisib, and further clinical studies should be done to confirm this result in patients.

## Introduction

**FIGURE 1:**
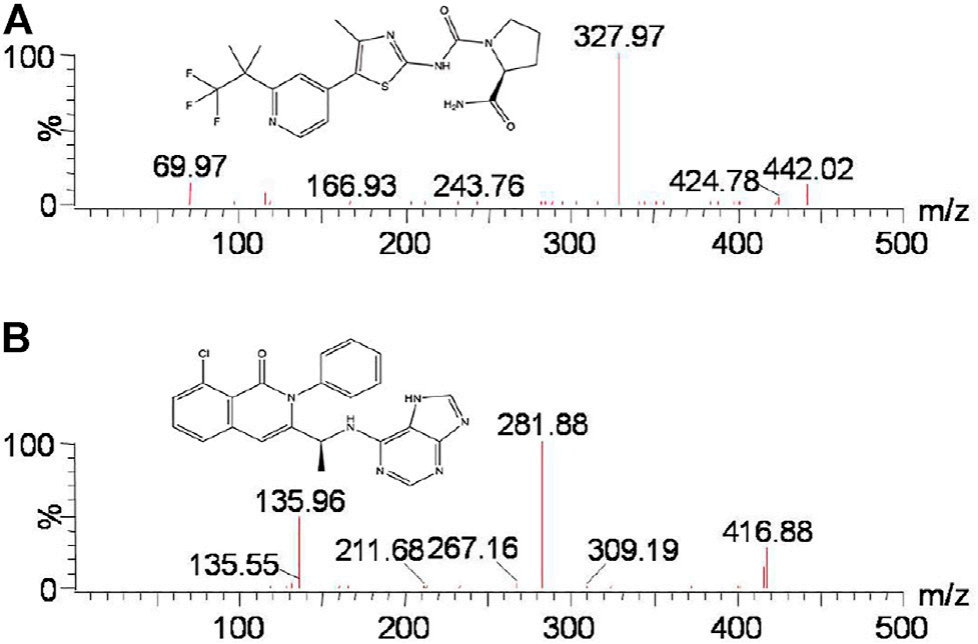
Mass spectra of alpelisib **(A)** and duvelisib **(**IS, **B**) in this study.

The phosphoinositide 3-kinase (PI3K) pathway has been the most common altered pathway in several cancers. Thus, drugs against different-level PI3K networks have been evaluated in clinical trials (Engelman, 2009; Thorpe et al., 2015). Alpelisib (BYL719, Figure 1A), an oral selective, small-molecule, and α-specific class I PI3K inhibitor, can selectively inhibit wild-type and mutant p110α approximately 50 times more effective than other subtypes (Brana and Siu, 2012; Furet et al., 2013; Chang et al., 2021). Several studies have demonstrated the efficacy of treating breast cancer as a single agent and combination therapy (Miller et al., 2010; Juric et al., 2018; Juric et al., 2019; Sharma et al., 2021). In addition, alpelisib, combined with fulvestrant, received approval in the United States to treat patients with breast cancer possessing hormone receptor and PIK3CA mutation, but without human epidermal growth factor receptor-2 (Andre et al., 2019; Markham, 2019; Andre et al., 2021).

Given that patients with cancer often take multiple drugs, whether the combination of alpelisib and other drugs would cause drug-drug interactions (DDIs) must be explored. CYP3A4 was proved to metabolize alpelisib *in vitro* experiment (James et al., 2015). Considering the importance of CYP3A4 to alpelisib metabolism, moderate and vigorous inhibitors of the CYP3A4 enzyme can alter the systemic exposure of alpelisib. Thus, it is essential to establish a quantitative method for alpelisib in biological fluids to investigate its pharmacokinetic characteristics and potential DDIs.

Some analytical methods have been reported to quantify alpelisib in biological matrices (De Buck et al., 2014;James et al., 2015;James et al., 2017). However, these analytical methods were not exhaustive and lacked sufficient experimental data to be reused in other laboratories. Recently, a technique using HPLC-FLD to quantify the plasma concentration of alpelisib from rats had been reported, having a long analytical time (> 10 min for each analyte) and complicated extraction procedure (Seo et al., 2021). Therefore, the objective of the present study was to establish a stable, simple, and hypersensitive ultra-performance liquid chromatography–tandem mass spectrometry (UPLC-MS/MS) assay to detect plasma alpelisib concentration in rats. Moreover, our newly developed method aimed to explore the impacts of diverse CYP3A4 inhibitors on alpelisib exposure and its pharmacokinetic alterations in the experiment of rats.

## Experiment

### Materials and Chemicals

Alpelisib, ketoconazole, itraconazole (purity >98%), formic acid, and duvelisib [internal standard (IS), purity >99%, Figure 1B] purchased from Beijing Sunflower Technology Development Co., Ltd. (Beijing, China) were used in this study. Both acetonitrile and methanol were LC grade and were supplied from Merck Company (Darmstadt, Germany). A Water Purification System from Milli-Q (Millipore, Bedford, USA) was used to acquire ultrapure water.

### Experimentation on Animals

Male Sprague–Dawley rats that we used, weighing 210 ± 10 g, were purchased from Wenzhou Medical University (Wenzhou, China), whose Laboratory Animal Center can provide animals. All experiment rats were kept under appropriate environment, including proper humidity, temperature, light conditions, rodent diet, and water. Before starting the experiment, all the rats were domesticated for 10 days under laboratory conditions to minimize their suffering. After obtaining approval from the Animal Protection and Use Committee of Wenzhou Medical University, animal experiments were carried out.

Alpelisib, itraconazole, and ketoconazole were all prepared with carboxymethyl cellulose sodium (CMC-Na) solution with the concentration of 0.5%. After 12 h of fasting but freely drinking, 18 rats were divided into three groups randomly (*n* = 6): 0.5% CMC-Na (group A), ketoconazole (20 mg/kg, group B), and itraconazole (20 mg/kg, group C). Alpelisib (30 mg/kg) was orally administered half an hour later. A blood sample of about 0.3 ml was then got from the tail veins of the rats at the time points of 0, 0.333, 0.667, 1, 1.5, 2, 3, 4, 6, 8, 12, 24, and 48 h and into 2.0-ml heparinized polythene tubes. Blood was then centrifuged at 4,000 × g at room temperature for 10 min, and the volume of 100 µl of plasma was collected immediately and kept at −80°C for further assay.

### Experimental Conditions

In this experiment, a Waters Xevo TQ-S triple quadrupole tandem mass spectrometer (Milford, MA, United States), connecting with a Waters Acquity UPLC I-Class system (Milford, MA, United States), was used to operate the assays. Before analysis, the mass spectrometric parameters were optimized and confirmed as follows: desolvation gas, 1000 L/h; capillary voltage, 2.0 kV; cone gas, 150 L/h; desolvation temperature, 600°C; and collision gas, 0.15 ml/min. The autosampler for analyzing all samples was kept at 10°C, along with 40°C for the temperature of the column. The measurement was implemented in positive ion mode using multiple reaction monitoring (MRM) with an electrospray ionization source (ESI). The specific parameters for alpelisib and IS are listed in Table 1. All the data were manipulated using the Quanlynx program with Masslynx 4.1 software (Milford, MA, United States).

**TABLE 1:** Specific mass spectrometric parameters and retention times (RTs) for alpelisib and IS, including cone voltage (CV) and collision energy (CE).

Analyte	Precursor ion	Product ion	CV (V)	CE (eV)	RT (min)
Alpelisib	442.02	327.97	30	20	1.32
IS	416.88	281.88	30	20	1.25

A C18 column from Acquity UPLC BEH (2.1 × 50 mm, 1.7 μm) was employed to separate alpelisib and IS in the plasma. The mobile phase had two components, namely, acetonitrile (A) and water possessing 0.1% formic acid (B). Linear gradient scheme was set and conducted as follows: 0–0.5 min, A/B = 10/90%; 0.5–1.0 min, A/B = 90/10%; 1.0–1.4 min, A/B = 90/10%; 1.4–1.5 min, A/B = 10/90%; and 1.5–2.0 min, A/B = 10/90%. The volume of each injection was 2.0 μl at a flow rate of 0.40 ml/min. The total run time was 2.0 min.

### Calibration Curve and Quality Control Samples

The compound was prepared with a proper amount of methanol to get alpelisib or IS stock solution with a concentration of 1.00 mg/ml. Then, quality control (QC) samples and calibration curve were respectively prepared by the corresponding working solutions diluted with methanol from the stock solution of alpelisib. Each concentration of working solutions with the volume of 10 µl was pipetted into blank plasma with the volume of 90 µl. Finally, both the calibration curve standards and QC samples for alpelisib were acquired with the concentration levels of 1, 5, 10, 50, 100, 500, 1,000, and 5,000 ng/ml, along with 2,800 and 4,000 ng/ml, respectively. The IS working solution for using in this study was 2.0 μg/ml by diluting with methanol. All prepared reagents were kept at −80°C for further use.

### Sample Treatment

Protein precipitation was performed by adding 300 µl of acetonitrile to 100 µl of plasma, followed by a 20 µl of IS working solution. The mixture was then centrifuged for 10 min at 13,000 rpm at the temperature of 4°C after mixing for 2.0 min. Finally, 100 µl of supernatant of each sample was drawn into a sample vial, where only 2.0 µl of the sample was used to inject into the autosampler for analysis.

### Bioanalytical Method Validation

A series of confirmatory experiments following the principles of Food and Drug Administration (FDA) based on the validation of bioanalytical assay (Xu et al., 2019; Tang et al., 2020; Center for Drug Evaluation and Research of the U.S., 2018.) was conducted: the lower limit of quantification (LLOQ), selectivity, carryover, calibration curve, precision and accuracy, recovery rate, dilution integrity, stability, and matrix effect.

### Statistical Analysis

In this study, Origin 9.0 software (Originlab Company, Northampton, MA, United States) was applied to determine the average concentration versus the time profile of alpelisib in plasma. The main pharmacokinetic parameters of alpelisib fitted with a non-compartmental model were calculated using DAS software (Drug and statistics, Version 2.0, Shanghai University of Traditional Chinese Medicine, China). Pharmacokinetics between groups was compared through one-way analysis of variance coupled with the Dunnett’s test using Statistical Package for the Social Sciences (version 17.0; SPSS Inc., Chicago, IL, United States). *p* < 0.05 is statistically significant.

### Results and Discussion in This Method

#### Assay Establishment and Optimization

In the present study, different chromatographic conditions and appropriate ion mode were tested to analyze alpelisib. Alpelisib (200 ng/ml) was injected into the mass spectrometer, and a higher signal-to-noise ratio was obtained in positive ion mode than in negative ion mode. Thus, the ESI in positive ion manner was selected to detect alpelisib. According to the predominant charge state and the fragment ions, the most suitable MRM ion pairs were *m/z* 442.02 → 327.97 for alpelisib and *m/z* 416.88 → 281.88 for IS. The retention time was short for both alpelisib and IS using a column of Acquity UPLC BEH C18 (2.1 × 50 mm, 1.7 μm). The aqueous phase with formic acid at volume percentage of 0.1% provided better peak shapes and higher responses. A quick and straightforward extraction method is also essential due to a sea of plasma samples involved in pharmacokinetics-associated experiments. We employed the method of protein precipitation with acetonitrile to precipitate proteins resulting from a higher recovery rate (> 89.5%) than other organic reagent (methanol, with <80% of recovery rate).

## Method Validation

### Selectivity and Carryover

**FIGURE 2:**
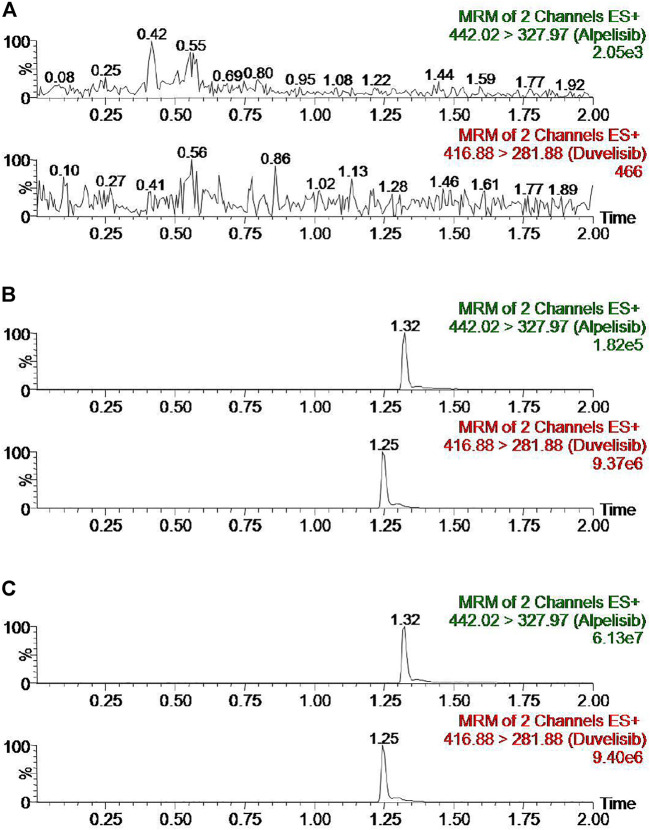
Representative chromatograms of alpelisib and IS in rat plasma: **(A)** blank plasma; **(B)** blank plasma spiked with standard solution at 10 ng/ml and IS; **(C)** sample obtained from a rat at 3.0 h after oral administration of alpelisib (30 mg/kg).

Figure 2 exhibits the representative MRM chromatogram peaks of six batches of blank rat plasma samples; the rat blank plasma sample pointed with the concentration of alpelisib (10 ng/ml) and IS, along with the actual plasma sample after the administration of alpelisib (30 mg/kg) in rats. Alpelisib and IS were identified at the retention periods of about 1.32 and 1.25 min, respectively, without detectable endogenous interference. Consequently, the method has good selectivity and specificity to determine alpelisib in plasma. In addition, no carryover was observed for either analyte or IS in rat plasma, because the peak area of the interference peak was less than 20% for the analyte and less than 5% for the IS in the LLOQ samples following injection of upper limit of quantification (ULOQ) samples.

### Standard Curve and LLOQ

The standard curve provided a perfect linearity within the scope of 1–5,000 ng/ml for alpelisib in rat plasma. The typical linear regression formula of alpelisib was obtained as follows: Y = 0.0819963 × X + 0.136784 (*r* ^2^ = 0.9996). The LLOQ was 1 ng/ml, possessing enough precision and accuracy (the results were shown in Table 2). Therefore, this method is sensitive to determine the plasma concentration of alpelisib in rats.

**TABLE 2:** The precision and accuracy of alpelisib in rat plasma (*n* = 6).

Analyte	Concentration (ng/ml)	Intra-day		Inter-day	
		RSD%	RE%	RSD%	RE%
	1	7.6	8.2	12.4	8.3
	2	6.2	5.0	8.4	2.7
Alpelisib	800	5.3	8.2	6.9	5.9
	4,000	4.1	2.8	4.3	1.1

### Accuracy and Precision

The accuracy and precision of alpelisib for inter-day and intra-day were quantified at LLOQ and three different QC concentrations on three separate days (*n* = 6), and the summary is presented in Table 2. The RE range of the intra- and inter-assay accuracy was 1.1% to ∼8.3%, with the RSD of the precision <12.4%. These outcomes indicated that the well-established assay had good accuracy, precision, and reproducibility and could be applied for the quantitative analysis of alpelisib in the plasma samples from rats.

### Matrix Effect and Extraction Recovery

Table 3 summarizes the extraction recovery and matrix effect of alpelisib. The recovery rate of alpelisib was from 89.5% to 94.9% at three QC concentrations in rat plasma, and the recovery of IS was 98.3%, indicating consistent recovery and precision. Similarly, the matrix effects ranged from 100.1% to 103.0%, and the matrix effect of IS was 99.8% to 107.4%. The IS-corrected matrix factor of alpelisib had an RSD of 6.9% or less, which met the acceptance criteria (not more than 15%), manifesting that the matrix effect had no significant influence on rat plasma.

**TABLE 3:** Recovery and matrix effect of alpelisib in rat plasma (*n* = 6).

Analyte	Concentration added (ng/ml)	Recovery (%)		Matrix effect (%)	
		Means ± SD	RSD (%)	Means ± SD	RSD (%)
	2	89.5 ± 3.2	3.6	103.0 ± 5.7	5.5
Alpelisib	800	90.9 ± 1.4	1.6	100.1 ± 2.3	2.3
	4,000	94.9 ± 2.5	2.7	96.7 ± 5.0	5.2

### Dilution Integrity

Dilution integrity ensures that dilution of a specimen with a concentration higher than ULOQ could result in an accurate quantification. Six replicates of 10-fold high concentration of QC samples (4,0000 ng/ml) with a 10-fold dilution were analyzed. The results showed that samples after dilution at 10-fold lied within the acceptable limits for accuracy and precision, which suggests that proper dilution is acceptable for the method.

### Stability

Stability experiments conducted in plasma samples at QC levels showed that alpelisib was durable and stable at ambient temperature for at least 6 h (short-term stability), in the autosampler (10°C) for 4 h thereafter extraction, three complete processes of freeze-thaw (−80°C to ambient temperature), and also at −80°C within 3 weeks (long-term stability).

### Pharmacokinetics

**FIGURE 3:**
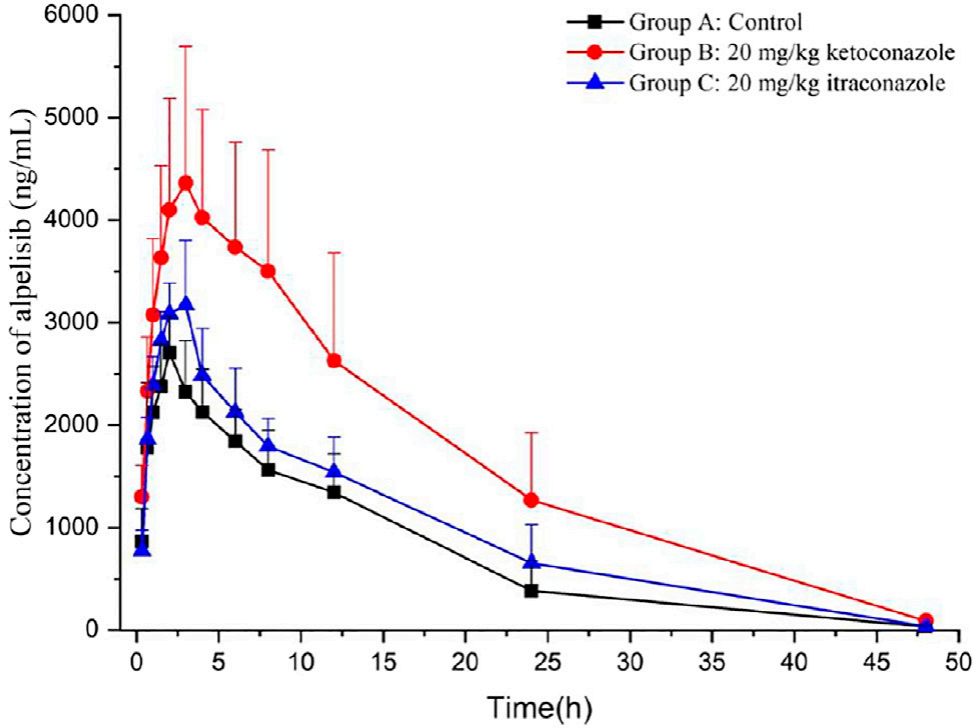
Mean plasma concentration-time curves of alpelisib in different treatment groups of rats: group A, the control group (0.5% CMC-Na); group B, a single-dose administration of ketoconazole (20 mg/kg); group C, a single-dose administration of itraconazole (20 mg/kg). (*n* = 6).

Through the novel developed bioanalytical assay based on UPLC-MS/MS techquine, the plasma concentrations of alpelisib in rats were detected successfully, and the pharmacokinetics from different groups were acquired. Figure 3 exhibits the average concentration versus time curves of alpelisib in each rat group after taking alpelisib (30 mg/kg) at a single oral dose. Table 4 sums up the essential pharmacokinetic parameters calculated under the mode of non-compartmental analysis.

**TABLE 4:** The main pharmacokinetic parameters of alpelisib in different treatment groups of rats: group A, the control group (0.5% CMC-Na); group B, a single-dose administration of ketoconazole (20 mg/kg); group C, a single dose administration of itraconazole (20 mg/kg). (*n* = 6, means ± SD).

Parameters	Group A	Group B	Group C
AUC_0→t_ (ng/ml·h)	36960.57 ± 916.91	80735.87 ± 23416.63*	46675.42 ± 10322.99*
AUC_0→∞_ (ng/ml·h)	37321.28 ± 6992.74	83183.86 ± 24255.31*	47063.20 ± 10431.90*
MRT_0→t_ (h)	10.75 ± 1.83	12.58 ± 1.76	11.53 ± 2.00
MRT_0→∞_ (h)	11.21 ± 1.94	13.10 ± 1.92	11.91 ± 2.12
t_1/2_ (h)	7.24 ± 0.69	8.77 ± 2.96*	7.88 ± 1.23
T_max_ (h)	2.08 ± 0.49	4.67 ± 1.97*	2.67 ± 0.52
CLz/F (L/h)	0.83 ± 0.15	0.39 ± 0.14*	0.67 ± 0.15
C_max_ (ng/ml)	2736.31 ± 378.34	4603.02 ± 1237.55*	3354.31 ± 407.83*

Compared with group A, *p < 0.05.

Ketoconazole and itraconazole have been extensively treating opportunistic fungal infections. It has been proved that ketoconazole is a potent CYP3A4 inhibitor (Gupta et al., 2014; Ogasawara et al., 2020) and so is itraconazole (Zhou et al., 2018; Barbour et al., 2019; Dai et al., 2019). Compared with group A, groups B and C raised the AUC_0→∞_ and C_max_ of alpelisib (*p* < 0.05), indicating that the total alpelisib systemic exposure increased. In addition, ketoconazole exhibits a more substantial inhibition on alpelisib metabolism than itraconazole. Therefore, the simultaneous use of alpelisib with a potent CYP3A4 inhibitor should be treated with extreme caution because the combination increases the exposure to alpelisib even more. If their combined use is unavoidable, then our data suggest that reducing the dose of alpelisib should be taken. Otherwise, the patient might suffer from some severe side effects caused by increased alpelisib plasma levels. The limitation of our research lies in the small number of rats used in the experiment.

## Conclusion

In the present experiment, a hypersensitive and accurate bioanalytical assay based on UPLC-MS/MS to determine alpelisib concentrations in plasma samples from rats was first established. The optimized method has been carefully verified under the FDA guidelines. Both ketoconazole and itraconazole exhibit inhibitory effects on the metabolism of alpelisib. While considering the complex and varied clinical factors of cancer patients, further human clinical trials on alpelisib should be investigated to confirm their accuracy interaction and be meaningful.

## Data Availability

The original contributions presented in the study are included in the article/supplementary material; further inquiries can be directed to the corresponding authors.
